# Cumulative impacts: thermally bleached corals have reduced capacity to clear deposited sediment

**DOI:** 10.1038/s41598-017-02810-0

**Published:** 2017-06-02

**Authors:** Pia Bessell-Browne, Andrew P. Negri, Rebecca Fisher, Peta L. Clode, Ross Jones

**Affiliations:** 1 Australian Institute of Marine Science, Townsville, QLD, and Perth, WA Australia; 2 The Oceans Institute and The Centre for Microscopy, Characterisation and Analysis, The University of Western Australia, Crawley, WA Australia; 3 Western Australian Marine Science Institution (WAMSI), Perth, WA Australia

## Abstract

The interaction between local, anthropogenic stressors, and larger scale regional/global stressors, is often used to explain the current poor condition of many corals reefs. This form of cumulative pressure is clearly manifested by situations where dredging projects happen to coincide with marine heatwaves that have caused coral bleaching. A key pressure associated with dredging is elevated sedimentation. In this study, 3 coral species (*Acropora millepora*, *Porites* spp. and *Turbinaria reniformis*), representing three common morphologies (branching, massive and foliose respectively), were experimentally induced to bleach by exposure to a temperature of 31 °C for 21 d. The corals were then subjected to a range of sedimentation rates (0, 11, 22 and 40 mg cm^−2^ d^−1^), and their sediment-rejection ability quantified after 1 and 7 successive sediment deposition events. Bleached corals were less capable of removing sediments from their surfaces, and sediment accumulated 3 to 4-fold more than on normally-pigmented corals. Repeated deposition resulted in a ~3-fold increase in the amount of sediment remaining on the corals, regardless of bleaching status. These results suggest that adaptive management practices need to be developed to reduce the impacts of future dredging projects that follow or coincide with elevated sea surface temperatures and coral bleaching events.

## Introduction

A well cited model for the current poor condition of many corals reefs is the interaction between local anthropogenic factors (e.g. coastal development, poor water shed management, pollution and overfishing), and larger scale regional and global factors (e.g. rising seawater temperatures and ocean acidification)^1–7^. This combination (and possible interaction) of local and global stressors represents one of the largest uncertainties for predicting environmental change^5, 8^. Conserving reefs in the face of predicted increases in seawater temperatures associated with climate change^3, 9–11^, and the pernicious threat of coral bleaching^12, 13^, is a significant challenge faced by regulatory agencies. Cumulative impacts and interactions of local and global stressors or drivers (see Côté, *et al*.^14^), can manifest themselves in many ways, and are epitomised by situations where dredging projects coincide with ‘natural’ warm-water coral bleaching events^15^.

Coral bleaching, or the dissociation of the coral-algal symbiosis, is a well-known stress response of corals^16–18^, and is frequently observed in response to periods of elevated water temperatures^3, 16, 19, 20^. Bleaching events can occur at multiple scales from local events (e.g. Jones^18^ and Jones^21^), to regional (mass bleaching) events^22, 23^, and even global bleaching events^3, 24, 25^. Bleaching events can occur rapidly (within days) from only short term acute periods of temporarily elevated water temperatures^21^, as well as in response to more chronic periods of warm water^26^. The loss of the symbionts is often associated with high levels of partial and whole colony mortality, and at the ecological level recovery of reefs impacted by severe bleaching occurs over decadal time frames^27^. Bleaching events differ considerably in severity and duration, and the sub-lethal response along with inter and intraspecific variability in bleaching of corals is commonly observed^28, 29^. While bleaching can lead to whole colony mortality, under some conditions heavily bleached (bone-white) colonies can regain their algal symbionts, and return to a normal pigmentation with no associated mortality^21^.

At a physiological level, recovery of algal symbionts can take many months to upwards of a year^26, 30–35^. This time frame is much longer than predicted from models based on the number of dividing *Symbiodinium* and duration of the division phase^34^. The extended time period needed to re-establish the symbiosis may be related to sorting processes within the tissues, i.e. the movement of newly divided algal cells into empty gastro-dermal cells^34^; however, it may also be due to tissue damage incurred during the bleaching process itself. In addition, heterotrophic food sources facilitate tissue repair following bleaching along with energy reserves, and limitations in either of these will also slow recovery^36–40^. Histopathological examinations of bleached corals sometimes show loss of architecture, various degrees of atrophy and necrosis, and disruption of the gastro-dermal layer^41–43^. Bleached corals have also been found to possess significantly depleted numbers of epidermal mucous secreting cells (mucocytes), compared to normally-pigmented corals^44, 45^. Corals exposed to elevated water temperatures also exhibit a six-fold higher release rate of undischarged cnidae, indicative of host cell necrosis or detachment^46^ during the bleaching process^47^.

Irrespective of the mechanism of recovery, bleached corals are in a compromised state, with decreased tissue thickness^48–50^, and in some cases low energy reserves^26, 51^. The reduced ability to acquire energy phototrophically can in some species be compensated for by increased heterotrophic feeding^36, 52^. However, most studies confer that bleached corals are in a weakened state and susceptible to range of different stressors, such as algal colonisation^53^, disease^54^, and periods of poor water quality^15^.

Concepts of multiple stressors acting synergistically, and of interactions between local anthropogenic factors and larger global factors, was highlighted recently with the Miami Harbor Phase III Federal Channel Expansion Project, which was conducted during a regional mass coral bleaching event^55, 56^. Similar situations have occurred in the inshore, central Great Barrier Reef ^21, 57^, and a series of dredging projects off the west coast of Australia in recent years^58^. The projects in Western Australia were large scale, capital dredging projects, which involved the removal of ~50 Mm^3^ of sediments from coral reef environments. These projects included the 2010–2011 Barrow Island project^59^, the 2007–2010 Pluto project^60^, and the 2013–2015 Wheatstone project^61^. These dredging campaigns each happened to coincide with a series of local and mass coral bleaching events^62–65^.

Dredging and dredging-related activities release sediments into the water column either from mechanical disturbance of the seabed, controlled overflow from hopper barges, or sediment disposal at offshore placement sites^66, 67^. There are many different cause effect pathways where sediment plumes from dredging activities can affect corals (recently reviewed by Jones, *et al*.^68^). Of these, one of the most significant is elevated sedimentation, as resuspended sediments deposit onto the sea floor, often leading to smothering of corals (see images in Foster, *et al*.^66^ and Jones, *et al*.^68^). In this study, we examine the consequences of coral bleaching on the tolerance of corals to poor water quality associated with dredging.

## Results

### Approach

Fragments of 3 coral species (*Acropora millepora*, *Porites* spp., and *Turbinaria reniformis*) were experimentally induced to bleach by exposure to elevated water temperatures. Sediment-rejection by these bleached corals was then tested against normally-pigmented ‘control’ corals, in a series of sediment deposition events up to a rate of 40 mg cm^−2^ d^−1^. Non-destructive techniques were used to monitor coral health throughout the sediment exposure period, including image analysis of coral colour and photochemical efficiency of algal symbionts. Corals were exposed to these sediment deposition events for 7 consecutive days, and at the end of each day any sediment accumulating on the surfaces was cleaned by water motion. The sediment-rejection ability of the corals was quantified after day 1 and then again at day 7 by determining the mass of sediment remaining on each coral.

### Effects of bleaching on coral survival, colour and physiology

**Figure 1:**
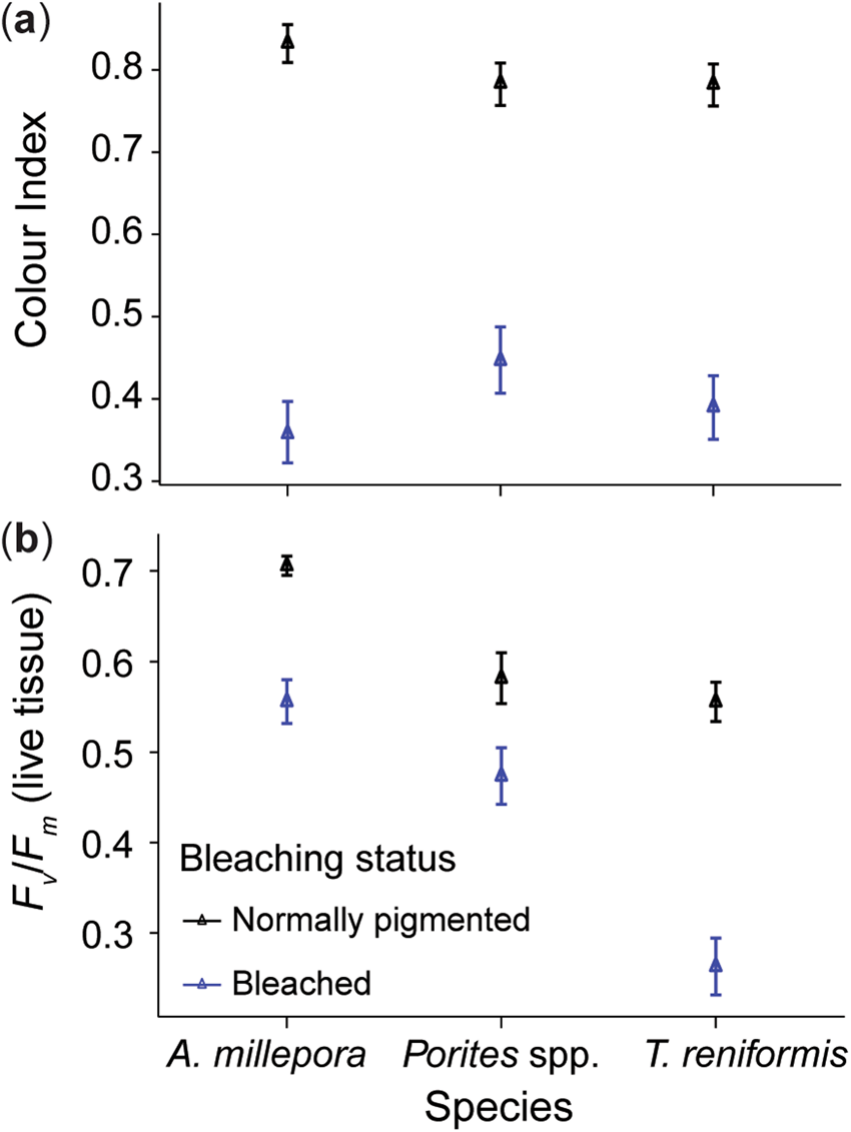
Differences in coral tissue colour and maximum quantum yield of the algal symbionts after the experimental bleaching. (**a**) Colour score index and (**b**) *F*_v_/*F*_m_ of bleached and normally-pigmented corals after exposure to 31 °C or 27 °C for 3 weeks and before the sediment deposition experiments. Average data is presented with SE, *n* = 48 fragments per bleaching status for each species.

Bleaching was induced in the corals by slowly raising water temperatures (0.5 °C per day for 8 d) and holding the temperatures at 31 °C for 3 weeks under a daily light integral of 8.6 mol photons m^−2^ d^−1^. Bleaching (tissue lightening) was observed after ~15−20 d in the 31 °C temperature treatment. No bleaching was observed in any of the corals held at 27 °C. For the branching *A*. *millepora*, paling occurred first on the upper light exposed surfaces, and towards the end of the exposure the upper surfaces were heavily bleached (bone white), and the lower (shaded) surfaces of the branches were pale in colour. The hemispherical *Porites* spp. also discoloured first on the upper more horizontal surface, with the edges of the colonies still retaining some pigmentation. Bleaching in *T*. *reniformis* was more uniform across the surface and the corals took on a pale yellow colouration (from animal pigments) as the bleaching progressed. There was no partial or whole-colony mortality resulting from the 31 °C temperature treatment, or in any of the corals held at 27 °C. The maximum quantum yields (*F*_v_/*F*_m_) of the bleached fragments were ~3, 2 and 6-fold lower in *A*. *millepora*, *Porites* spp., and *T*. *reniformis* respectively, and bleached corals had ~50% lower colour index scores than the normally-pigmented fragments for all species (Fig. 1).

### Effects of pulsed sediment on coral survival, colour, physiology and sediment clearance

**Figure 2:**
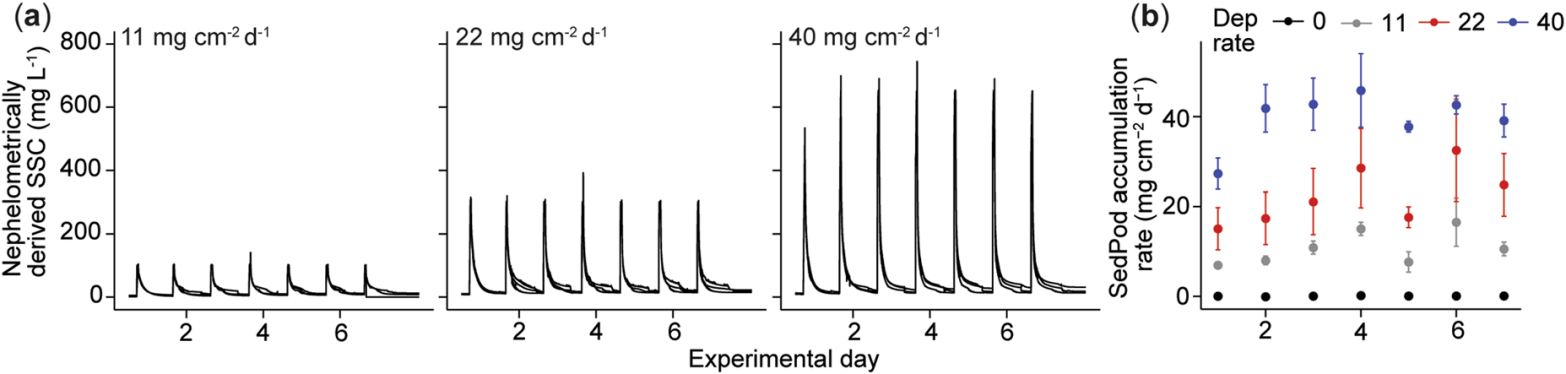
Suspended sediment concentrations (SSCs) and sediment deposition rates in the experimental tanks. (**a**) Nephelometrically-derived suspended sediment concentrations (mg L^−1^) in each tank for the 7 day exposure period showing resuspension of sediment each afternoon (4:00 pm) followed by a decrease in SSCs as sediment fell out of suspension on the corals and SedPods each night, (**b**) Sedimentation (SedPod accumulation rate, mean ± SE, *n* = 3) after each of the deposition events across the 4 deposition rate treatments of 0, 11, 22 and 40 mg cm^−2^.

The bleached and normally-pigmented corals were exposed to temporarily high suspended sediment concentrations (SSCs), and water motion in the tanks was subsequently stopped to allow the sediments to settle out of suspension and onto the corals. Settlement of sediment in the tanks occurred rapidly, with most sediment dropping out of suspension in the first 1–2 h after the pumps were turned off each day (Fig. 2). The amount of sediment accumulating on the flat, concrete filled PVC cylinders (SedPods) averaged 0.05 ± 0.03, 10.8 ± 3.7, 22.4 ± 2.4 and 39.6 ± 2.3 mg cm^−2^ (mean ± SE, *n* = 3) over the 7 successive deposition events (Fig. 2).

There was no whole colony mortality in response to the sediment exposures and the only partial mortality occurred in a bleached *Porites* spp. fragment (which suffered 26% partial mortality in the 11 mg cm^−2^ d^−1^ treatment), and a bleached fragment of *T*. *reniformis* (which suffered > 5% mortality in the 22 mg cm^−2^ d^−1^ treatment).

During the sediment exposures, all species had expanded and partially expanded polyps irrespective of bleaching status. Mucus secretion was commonly observed in *T*. *reniformis* and *Porites* spp., but less so in *A*. *millepora*. All corals appeared to corral and collect the sediments into mucus-laden patches with well–defined, discrete, edges. The patches were then slowly moved under muco-ciliary transport to the sides of the colonies and ultimately shed. Occasionally, sediments patches became stuck in local depression or concave features of the surface and remained trapped there. For the *A*. *millepora* similar trapping of sediment was occasionally observed at the junction point of neighbouring branches.

**Figure 3:**
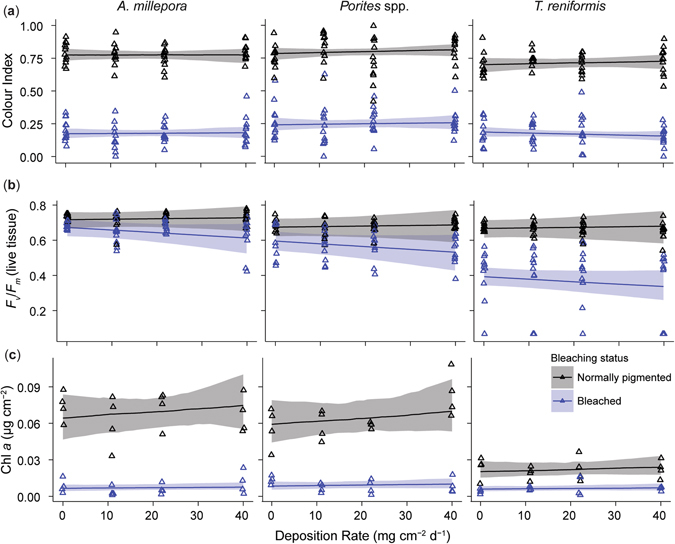
Physiological health parameters of bleached and normally-pigmented corals after 7 consecutive deposition events. (**a**) Colour index, (**b**) Maximum quantum yield (*F*_v_/*F*_m_), and (**c**) Chl *a* concentrations (µg Chl *a* cm^**−**2^) of *A*. *millepora*, *Porites* spp. and *T*. *reniformis* fragments across 4 deposition rate treatments for both normally pigmented (black) and bleached (blue) fragments after 7 consecutive deposition events. Raw data (triangles) is presented with modelled relationships (lines) and 95% confidence intervals (ribbons).

The colour index of bleached and normally-pigmented corals differed considerably for all three species, while there was limited apparent impact of sediment treatment (Table 1, Fig. 3). The best fit model suggested that colour index was driven by an interaction between bleaching status, species and time, with an additive impact of sediment treatment having a lower model weight than when sediment was included (Table 1, Fig. 3). Chl *a* was best explained by a model including an interaction between bleaching status and species (Table 1, Fig. 3). At the end of the experiment Chl *a* concentrations differed between normally-pigmented and bleached corals, with substantially less Chl *a* present in bleached fragments, with this consistent across all three species, while the smallest difference was apparent in *T*. *reniformis* (Fig. 3). Similar to colour index, there was no evidence for an impact of sediment deposition treatment on Chl *a* concentrations (Fig. 3), with a model not including sediment having a higher model weight (Table 1). *F*_v_/*F*_m_ also differed between normally-pigmented and bleached corals, and this effect was dependent on species, time and sediment (Table 1). While *F*_v_/*F*_m_ remained stable across deposition treatments for the normally-pigmented corals, *F*_v_/*F*_m_ values were lower in higher sediment treatments in bleached corals of all species (Fig. 3).

**Table 1:** Top model fits (generalised linear mixed model) for colour index, Chl *a* concentrations, maximum quantum yield (*F*_v_/*F*_m_) and deposited sediment for each of the fixed factors, inducing bleaching status (ble.), species (spp.), time and sediment deposition treatment (sed).

Parameter	Model	*n*	AICc	δ AICc	AICc weight	R^2^
Colour index	**ble**.** × spp**.** × time**	**15**	**933**.**6**	**0**.**0**	**0**.**33**	**0**.**83**
	ble.** × **spp.** × **time + sed.	16	935.3	1.7	0.14	0.84
	ble.** × **spp.** × **time + spp.** × **time + ble.** × **time + ble.** × **spp. + sed.	17	936.0	2.4	0.11	0.84
	spp.** × **time + ble.** × **time + ble.** × **spp.	13	936.0	2.4	0.10	0.83
	ble.** × **spp.** × **time + spp. x time + sed.** × **time + ble.** × **time + ble.** × **spp.	17	937.4	3.9	0.05	0.84
	spp.** × **time + ble.** × **time + ble.** × **spp. + sed.	14	937.8	4.2	0.04	0.83
Chl *a*	**ble**.** × spp**.	**9**	**−650**.**4**	**0**.**0**	**0**.**41**	**0**.**93**
	ble.** × **spp. + sed.	8	−649.9	0.5	0.32	0.93
	ble.** × **spp. + ble.** × **sed.	10	−646.9	3.0	0.10	0.93
	sed.** × **spp. + ble.** × ** spp.	11	−645.7	4.2	0.06	0.93
	ble.** × **sed.** × **spp.	14	−643.9	6.0	0.02	0.94
	sed. x . spp. + ble.** × **spp. + ble.** × **sed.	12	−643.1	6.8	0.02	0.93
*F*_*v*_/*F*_*m*_	**ble**.** × spp**.** × time + ble**.** × sed**.	**17**	**798**.**7**	**0**.**0**	**0**.**23**	**0**.**63**
	ble.** × **spp.** × **time + ble.** × **sed. + spp.** × **sed.	19	800.0	1.3	0.12	0.64
	ble.** × **spp.** × **time + sed. x time + ble. x spp.	18	800.3	1.7	0.10	0.63
	ble.** × **spp.** × **time + sed. x time + sed. x spp.	19	801.4	2.7	0.06	0.64
	ble.** × **spp.** × **time + ble.** × **sed.** × **spp.	20	801.6	3.0	0.06	0.64
	sed.** × **spp.** × **time + ble.** × **spp.** × **time	21	801.6	3.0	0.05	0.64
Deposited sediment	**ble**.** × spp**.** × time + sed**.** × time + sed**.** × spp**.** + sed**.** × ble**.	**20**	**787**.**5**	**0**.**0**	**0**.**39**	**0**.**99**
	ble.** × **spp.** × **time + ble.** × **sed.** × **time	21	789.3	1.9	0.15	0.99
	ble.** × **spp.** × **time + ble.** × **sed.** × **spp. + sed.** × **time	22	790.8	3.3	0.07	0.99
	spp.** × **time + sed.** × **time + sed.** × **spp. + ble.** × **time + ble. x sed.	16	791.0	3.5	0.07	0.99
	spp.** × **time + sed.** × **time + sed.** × **spp. + ble.** × **time + ble.** × **spp. + ble.** × **sed.	18	791.1	3.7	0.06	0.99
	sed.** × **spp.** × **time + ble.** × **spp.** × **time	22	791.5	4.0	0.05	0.99

Shown are the fitted model, number of parameters (*n*), Akaike information criterion (AICc), δ AICc, model weights, and R^2^ values. The model with the fewest parameters within 2 AICc is considered the most parsimonious, and therefore the best model. Selected best models are shown in **bold**.

**Figure 4:**
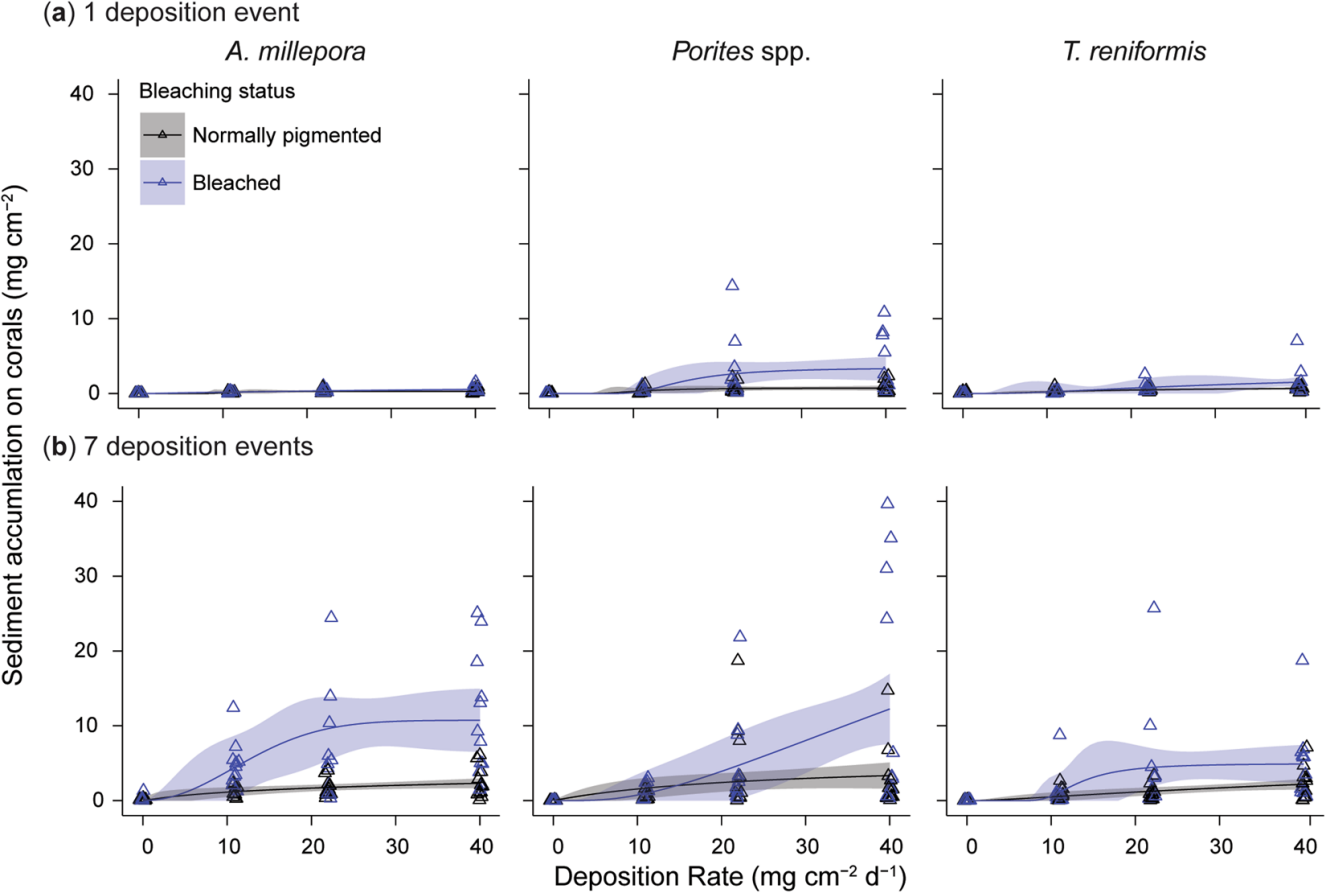
Sediment accumulation on bleached and normally-pigmented corals. The amount of sediment (mg cm^**−**2^) accumulated on each fragment of *A*. *millepora*, *Porites* spp. and *T*. *reniformis* across 4 deposition rate treatments for both normally-pigmented (black) corals and bleached (blue) fragments after: (**a**) 1 deposition event, and (**b**) a further 6 deposition events. Raw data is presented (triangles) with modelled values (lines) and 95% credible intervals (ribbons).

Normally-pigmented corals were effective at removing sediment from their surfaces, and at the highest sedimentation level the amount of sediment remaining was 0.6 ± 0.2 mg cm^−2^ (mean ± SD, range 0.3–0.7 mg cm^−2^, *n* = 36), or 2.1% of the accumulation rate measured on the SedPods on that day (Supplementary Table S1, Fig. 4). The difference between the sediment mass on the corals and SedPods is due to the self-cleaning ability of the corals. Sediments were washed off the corals each day (during the re-suspension process), and after the last of the 7 consecutive daily deposition events, the amount of sediment remaining on the surfaces of the highest sedimentation level, was much higher, being 2.5 ± 0.3 mg cm^−2^ (mean ± SD, range 2.3–2.8 mg cm^−2^, *n* = 36), or 6.4% of the accumulation rate measured on the SedPod on that day (Supplementary Table S1, Fig. 4). Overall, for the normally-pigmented colonies, 3.1-fold more sediment remained on the surface of the corals after the 7^th^ deposition event than after the first.

Bleached corals were less effective at removing sediment from their surfaces, and at the highest sedimentation level, the amount of sediment remaining was 1.8 ± 1.4 mg cm^−2^ (mean ± SD, range 0.5–3.3 mg cm^−2^, *n* = 36), or ~6.5% of the accumulation rate measured on the SedPod on that day (Supplementary Table S1, Fig. 4). After the last of the 7 consecutive daily deposition events, the amount of sediment remaining on the surfaces of the highest sedimentation level, was much higher at 9.3 ± 3.9 mg cm^−2^ (mean ± SD, range 4.9–12.3 mg cm^−2^, *n* = 36), or 24% of the accumulation rate measured on the SedPod on that day (Supplementary Table S1, Fig. 4). Overall 3.7-fold more sediment remained on the surface of the corals after the 7^th^ deposition event than after the first.

The combined effects of the reduced capacity to self-clean with time and lower sediment rejection ability of bleached corals differed between species. For bleached *T*. *reniformis* and the *Porites* spp. 2.3 to 2.6-fold more sediment remained on the corals after the last deposition event than the first. For bleached *A*. *millepora*, 15-fold more sediment remained on the corals after the last deposition event than the first (Supplementary Table S1, Fig. 4). The amount of remaining sediment on each fragment (inability to clear sediments), was best described by a complex model involving a three-way interaction between bleaching status, species and time as well as two-way interactions between sediment deposition and species, along with time and bleaching status (Table 1, Fig. 4). Given the high order interactions with deposition, we fitted separate dose response relationships at two times (after 1 deposition and after 7 deposition events), for each species and for both bleached and normally pigmented corals.

Effect concentrations of both 10 and 50% (EC_10_ and EC_50_ respectively) impacts on clearance ability were calculated for bleached colonies of each species after 7 deposition events (Fig. 4). A sediment deposition rate of 40 mg cm^−2^ d^−1^ was not sufficient to reduce the clearance ability of any species while normally pigmented after either 0 or 7 deposition events (Fig. 4). Bleached *A*. *millepora* demonstrated a 10% reduction of clearance ability at 5 ± 4 mg cm^−2^ d^−1^, and a 50% reduction at 12 ± 4 mg cm^−2^ d^−1^, both after 7 deposition events. The 10% impact on clearance in bleached *T*. *reniformis* after 7 deposition events was 10 ± 3 mg cm^−2^ d^−1^, while the 50% impact was observed at 13 ± 6 mg cm^−2^ d^−1^. After 1 deposition event the 10% impact on clearance for *Porites* spp. was 11 ± 4 mg cm^−2^ d^−1^, while a 50% impact was observed at 16 ± 6 mg cm^−2^ d^−1^. No effect concentration could be determined for *Porites* spp. after 7 deposition events as the amount of sediment accumulating on the fragments was still increasing at the highest sediment deposition treatment (Fig. 4b).

**Figure 5:**
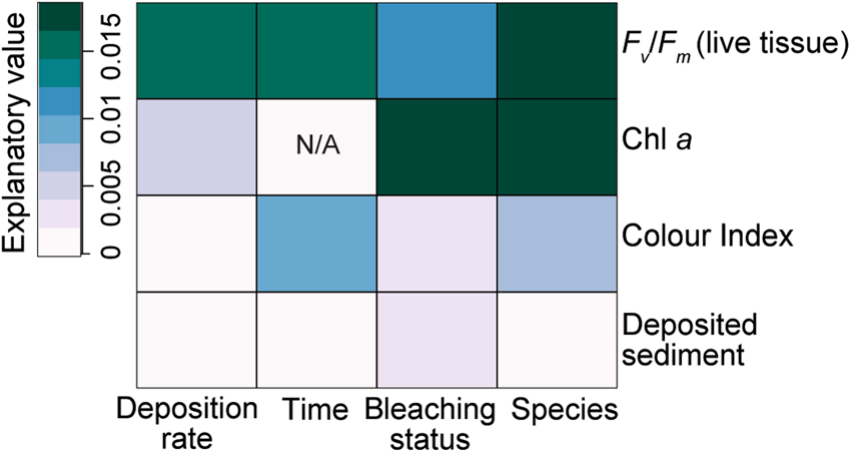
Explanatory value of each of the fixed factors included in the models, including deposition rate treatment (mg cm^−2^), Time (d), Species and bleaching status (normally pigmented or bleached), with darker colours indicating increased importance of that variable. Explanatory value was calculated simply as the additional R^2^ obtained when a variable was included in a complete interaction model, compared to a model excluding just that variable. This in effect represents the unique variance explained by each predictor, rather than the overall R^2^ explained by individual best models, as presented in Table 1.

Based on relative explanatory values of the fixed predictors, species was the most important factor influencing *F*_*v*_/*F*_*m*_, followed closely by sediment treatment and time, and then lastly bleaching status, however these were all strong influencers (Fig. 5) Colour index was most influenced by time, followed by species, bleaching status, and finally sediment treatment, which had a limited effect (Fig. 5). Chl *a* concentrations were most influenced equally by bleaching status and species (although time was not included in these models because this was only measured at the end of exposure), followed by sediment deposition rate (Fig. 5). Lastly, the amount of deposited sediment remaining on coral fragments was most strongly driven by bleaching status (Fig. 5).

**Figure 6:**
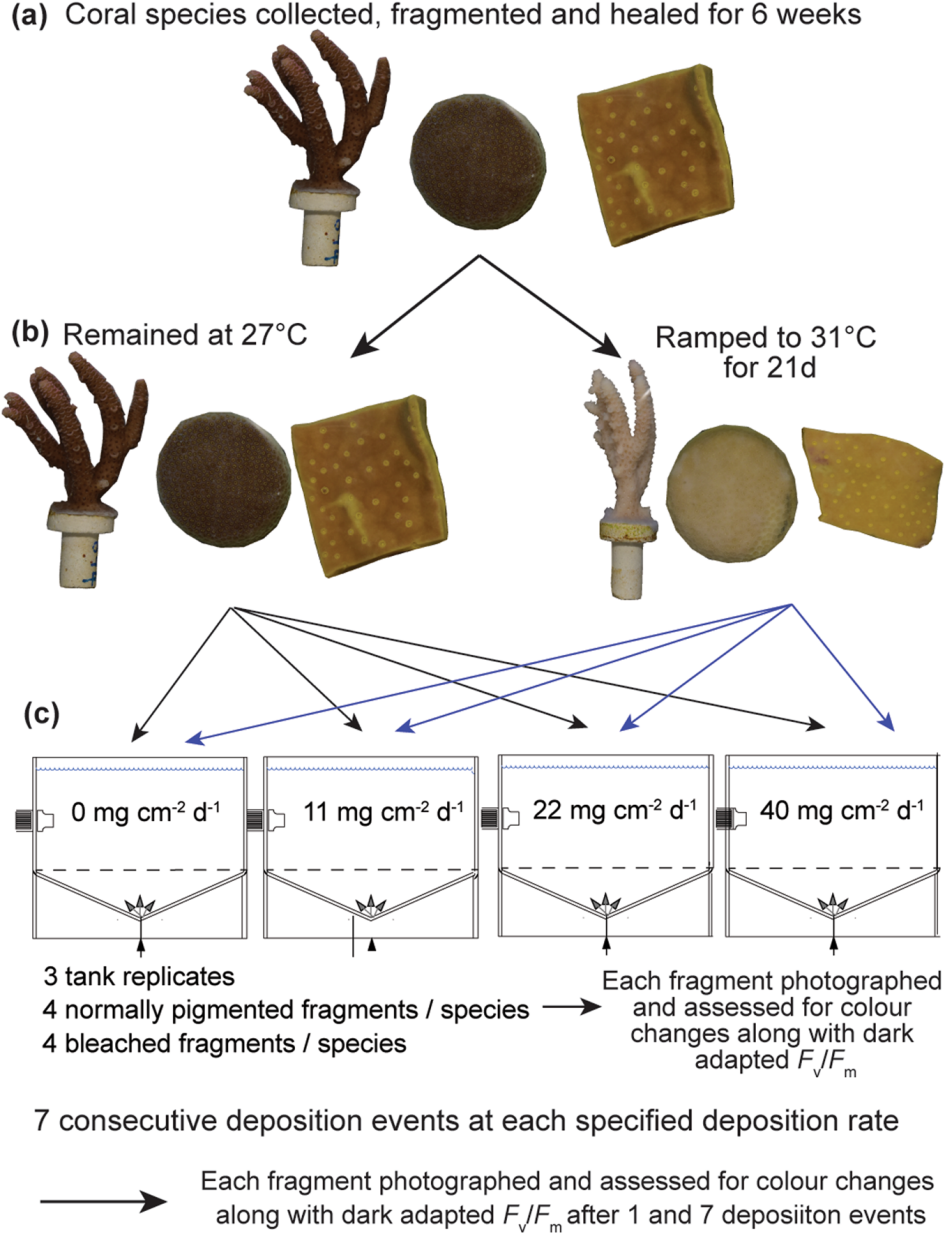
Conceptual diagram of the steps involved in the experiment, including (**a**) the initial healing process, (**b**) bleaching of corals, (**c**) subsequent sediment deposition exposures and health parameters that were assessed at different stages of the experiment.

## Discussion

The data from this study provides clear evidence that thermally bleached corals have considerably reduced capacity to clear sediments from their surfaces compared to normally-pigmented corals. The pattern was similar across three growth forms (branching, massive, and foliose), from three common and widely distributed coral species. Sedimentation is considered one of the most widespread, human-induced perturbations on reefs^69^, and a key causal pathway associated with mortality of corals close to dredging activities^68, 70–72^. This interaction between coral bleaching and sedimentation pressures clearly represents a consideration for resource managers in the regulation of more manageable local, anthropogenic stressors in the face of much wider-scale, and essentially uncontrollable issues^15^, associated with periods of elevated water temperature resulting from a changing climate.

Before the sediment exposure experiment, corals were artificially bleached by heat stress. The time-course and patterns of bleaching are consistent with the current understanding of the bleaching phenomenon based on both field and laboratory experiments^73^. Bleaching was induced by slowly raising water temperatures to 31 °C and exposing the corals for 3 weeks under a daily light integral of 8.6 mol photons m^−2^ d^−1^. For the mid-shelf reefs of the GBR, at around 18°S, where the corals were collected, the maximum average daily temperatures typically range from 28–30 °C^73^. The 31 °C temperature only marginally exceeds the normal range, although the combination with time (21 d exposure) greatly exceeds temperatures that the corals have previously encountered, and is equivalent to ~4 degree heating weeks (DHW). Such a DHW exposure is generally anticipated to result in significant coral bleaching and this exposure resulted in the dissociation of the symbiosis in all species^74^. Maximum potential quantum yields (dark-adapted *F*_v_/*F*_m_) of the algal symbionts were significantly reduced by the temperature/light combination, and corals preferentially bleached first on their upper, light exposed surfaces. These results and observations are consistent with a photoinhibition model of coral bleaching^3, 44, 75–80^.

Corals have a range of mechanisms to clean their surface of sediments primarily involving muco-ciliary transport, hydrostatic inflation and tentacle movement^72, 81–85^. These energy intensive ‘active’ processes work in combination with ‘passive’ forces associated with gravity and flow to keep the upper sunlight exposed surfaces sediment-free in all but extreme cases of deposition. If sediments cannot be removed from the surface at a rate equivalent to the deposition, sediment can then build up and smother coral tissue, reducing feeding, solute (gas) exchange, and light transmission to the algal symbionts^86^. If covered with several millimetres of organic rich sediment mortality can occur quickly, in a matter of days^87^.

For the most part the corals were able remove all sediments up to the highest deposition rate tested of 40 mg cm^−2^ d^−1^. For contextual purposes deposition rates of up to 50 mg cm^−2^ d^−1^ have recently been measured during a high wind and wave event on a inshore turbid reef system of the central Great Barrier Reef ^88^. These maximum deposition rates occurred during a natural resuspension event and a period of extreme turbidity, where wind-speeds exceeded the 95^th^ percentile for the local area, and SSCs exceeded 100 mg L^−1^ ^88^. Under less extreme conditions and when SSCs ranged from a more typical < 1–28 mg L^−1^, deposition rates in the naturally turbid reef system averaged only 8 ± 5 mg cm^−2^ d^−1^ ^88^. This suggests that the high sediment deposition rates investigated here are likely to be associated with either very extreme natural turbidity events or in close proximity to dredging activities.

The corals in this study cleaned their surfaces of sediments using muco-ciliary transport, which is commonly regarded as the primary mechanism whereby corals can move fine silts and clays from their surfaces. The fluidic mucus is produced from epithelial secretory cells, mucocytes^89, 90^, and because of its adhesive characteristics can agglutinate settled sediment. Mucus-entangled sediment^81^ was manipulated by co-ordinated ciliary movement (metachronal waves), and the movement of sediment invariably occurred down the face of sub-horizontal, inclined planes until reaching the colony edge where is was subsequently shed. The normally-pigmented corals were highly efficient at removing sediments, and in the highest sedimentation rate the amount of sediment remaining on the tissues was only ~2% of the amount that settled on the inert, flat surface of the SedPods. This difference is due to the self-cleaning, sediment rejection ability of the corals, which actively removed sediment from the colony surface. For both the bleached and normally pigmented corals there was a broadly similar (3.1 and 3.7-fold respectively) increase in the amount of sediment remaining on the tissues after the seventh successive daily deposition than the first, indicating a reduction in self-cleaning capability with time. Where the two types of corals (bleached and normally-pigmented) differed was in the absolute levels of deposited sediment on their tissue, which was typically 3 to 4-fold higher on bleached than the normally-pigmented corals at the highest sedimentation rate (40 mg cm^−2^ d^−1^). The combined effects of the lower sediment rejection ability of bleached corals and the more general loss of self-cleaning ability with time meant the amount of sediment on the bleached corals after the last deposition event averaged ~24% of the amount that settled on the SedPods. For the most sensitive species, *A*. *millepora*, this amounted to a 15-fold decrease in the amount of sediment that could be cleared from the surface.

There are numerous plausible mechanisms for these two effects. Histological studies have demonstrated an absence of mucocytes in the epithelium of an experimentally bleached (heat-stressed) coral (*Stylophora pistillata*), and negligible quantities of mucus in the deeper gastro-dermal layer^44^. Similarly, Piggot, *et al*.^45^ showed that in field-collected corals, those that have bleached through heat stress have greatly reduced densities of epithelial mucocyte cells, even though the density of mucocytes increases with increasing water temperature. Mucocyte numbers and mucus production was not quantified in this study, but reduced mucocyte density in bleached corals seems plausible. Mucus production comes at an energetic cost to corals^91, 92^, and numerous studies have qualitatively suggested increased mucus production in sediment-exposed corals^72, 93, 94^ and repeated exposure to sediment deposition events could also exhaust mucus production. Stafford-Smith^94^ argued a counterpoint, that it is ciliary transport that becomes exhausted and that cannot be maintained for long period, citing the ability of some corals to produce mucus in response to sediment influx long after sediment rejection slows down. Recent studies have shown that cilia beating is only a negligible fraction of the corals metabolic budget^95^; but possible changes under sediment influx are unknown^68^.

Ultimately the energy for mucus production and ciliary transport would come from phototrophic or heterotrophic sources, both of which would be affected by sediment smothering. For phototrophy, both the loss of algal symbionts, and the reduced light availability to remaining algae by a thin layer of sediment would decrease energy availability^92^, as light transmission is <1% through a ~2 mm deposit of very fine, silt size sediment^86, 87^. Corals were not veneered by sediments in this experiment as they corralled the sediments into discrete patches, but under these patches light availability would be limited, thus impacting upon photosynthesis and potential recovery from the bleached state. Corals rely on heterotrophic food sources and energy stores during recovery from bleaching for at least 11 months^36–38, 40^. This suggests that the ability to re-establish the coral-*Symbiodinium* symbiosis depends on the health of the coral host and these alternative energy sources will be vital in the recovery of corals. In addition, some corals have been found to obtain nutritional value from sediment^96^, and this may aid in recovery from a bleached state when exposed to elevated sediments, however, this would only occur at low sediment concentrations as SSCs above 30 mg L^−1^ have been found to reduce coral feeding rates^97^. In a natural setting, sediment related stress may be greater than reported here, where sediments have been manually cleared from corals on a daily basis, as while periodic sediment removal may occur naturally during storm events, it seems unlikely this would occur on a daily frequency. Due to this increased accumulation of sediments on coral tissues would likely occur, leading to mucus sheet formation^98^ and potentially necrosis^87^. The reduced capacity of bleached corals to removed sediment will mean that such effects will be substantially greater in corals compromised by thermal stress.

In summary, this study has demonstrated that thermally bleached corals have substantially reduced ability to clear deposited sediment, and that this ability is further reduced following consecutive days of sediment deposition. The impacts of this reduction in clearance ability is likely to become exacerbated when combined with increased ocean acidification, another key impact resulting from climate change. The likelihood of a marine heatwave (and subsequent coral bleaching event) coinciding with dredging projects has unfortunately reached a point where explicit consideration needs to be given regarding management practices. Maintenance dredging typically occurs over a few weeks to months, and the timing of the activities can be reasonably predicted and planned in advance^99^. Avoiding dredging when bleaching could occur (i.e. summer maximum temperatures) seems a practical approach. This management practice is similar to concepts associated with ‘environmental windows’, which involve avoidance of times of the year or particular sites, where key species, ecological communities or critical processes may be particularly vulnerable to pressures from dredging^100, 101^. For capital dredging projects which can often occur over extended periods^102^, contingency plans in the event of a warm-water bleaching event could be developed before dredging commences. Plans could include defining the scale and intensity of an event and a series of practical and achievable courses of action to ensure minimal impacts and maintain acceptable levels of impact. Appropriate courses of action may include relocation of dredges, altering overflow plans, use of different dredge material placement sites, the use of more conservative water quality thresholds (for managing projects), and possibly cessation of all dredging activities. Several of these approaches were employed on the Great Barrier Reef in a dredging program in 2000–2001^21, 57^. From an environmental and management perspective, the significance of sub-lethal bleaching is that the symbiosis is dissociated, and the time for it re-establish may be much longer than the acute physiological effects alone. This serves to amplify the significance of what may be a short-term initial stress, increasing the potential for additional impacts from local, anthropogenic activity (i.e. cumulative impacts).

## Materials and Methods

Experiments were conducted with 3 hard coral species, *Acropora millepora* (Ehrenberg 1834), *Porites* spp. and *Turbinaria reniformis* (Bernard 1896), representing branching, massive and foliose morphologies respectively. Due to difficulty identifying *Porites* spp. underwater (they have small and variable corallites^103^) a mixture of two species (*P*. *lutea* and *P*. *lobata*) were included. All corals were collected between 3–10 m from the Rib, Trunk and Davies Reef lagoons (mid-shelf reefs centrally located in the central Great Barrier Reef, GBRMPA permits G12/35236.1 and G13/35758.1). For *A*. *millepora*, 8 colonies were collected by hand, 14 *Porites* spp. colonies were cored with a pneumatic drill, and *T*. *reniformis* were collected from an extensive thicket ~5 by 10 m where individual colonies were unable to be identified. 10 fragments were collected from each *A*. *millepora* and *T*. *reniformis* colony, while 6 were collected from each *Porites* spp. colony. Colonies that were free of biofouling and diseases were fragmented into replicates of ~7 cm^2^. Fragments were then glued onto aragonite coral plugs, with genotypes randomly allocated between 6, 200 L flow-through (receiving new water at a rate of 31 L h^−1^ = 3 water turnovers d^−1^) holding tanks in the National Sea Simulator (SeaSim) at the Australian Institute of Marine Science (AIMS, Townsville, Australia), for 6 weeks to recover from the collection and preparation procedures (Fig. 6a). Light above each tank was provided by four AI Hydra FiftyTwo™ HD LED lights (Aquaria Illumination, IA, US), which generated even illumination with an equal mix of white, blue, and red light. During the holding period, corals were exposed to a 12 h light:dark (L:D) cycle comprised of a 6 h period of gradually increasing light in the morning (06:00–12:00 h), to a maximum midday instantaneous light level of 400 μmol photons m^−2^ s^−1^ followed by 6 h of gradually decreasing light in the afternoon (12:00–18:00 h). Over the course of the day the corals experienced a daily light integral (DLI, total summed Photosynthetically Active Radiation [PAR, 400–700 nm]) of 8.6 mol photons m^−2^. Light intensity was measured within tanks at the same depth as the coral fragments using an Underwater Spherical Quantum Sensor (Licor LI-193).

Prior to the start of the sediment clearance experiments, half the coral replicates were induced to bleach by exposing them to elevated water temperatures (Fig. 6b). To induce bleaching water temperatures in four of the six holding tanks was incrementally raised from 27 °C to 31 °C at a rate of 0.5 °C per day for 8 d. Corals were then maintained at 31 °C (or 27 °C) for 3 weeks. Light exposure remained the same as during the holding period. Coral bleaching was observed by the 3^rd^ week of exposure, and was examined throughout the bleaching period by visual assessment, and the use of a colour index (see below). The maximum quantum yield (*F*_*v*_/*F*_*m*_) of symbiotic dinoflagellate algae within the live tissue of each coral fragment was also measured using a mini-PAM fluorometer (Walz, Germany) (see below).

Once the corals had been bleached, their ability to clear upward facing surfaces of sediment was compared with normally-pigmented corals, by exposure to a series of sedimentation events of different intensities (Fig. 6c). All experiments were conducted in clear PVC tanks filled with 100 L of 0.4 µm filtered seawater (as not to introduce additional uncontrolled sediment sources) pumped into each tank at 400 mL min^−1^ to ensure 5.8 complete water changes per day, see Bessell-Browne, *et al*.^104^ for details of the experimental system. Three replicate tanks were used to enable potential tank-effects to be assessed. Water temperature was maintained at 27 ± 0.5 °C and salinity was 33 ± 0.5‰. Light above each tank was provided by two AI Hydra FiftyTwo™ HD LED lights (Aquaria Illumination, IA, US). Corals were exposed to a daily light integral (DLI) of 8.6 mol photons m^−2^ using the same light regime as the bleaching experiment described previously. Coral genotypes were randomly allocated between the experimental tanks with 4 bleached and 4 normally pigmented corals assigned to each tank.

Corals were placed in the tanks on a fibre reinforced plastic grating (false bottom floor), 25 cm below the water surface. The sediment used for deposition events had a mean grain size of 30 µm, and this was used as it has been found to be the most common grain size suspended and subsequently deposited during dredging campaigns^68^. Deposition events were created in each tank by raising the SSCs to 100, 300 and 650 mg L^−1^ (nephelometrically derived SSCs), and then turning the pumps off and allowing the suspended sediment to settle out of suspension overnight past the grating holding the corals. At the end of the day (4.00 pm), the pumps were turned on again resuspending all sediment that had settled on the bottom of the tank, grating and the corals. Repeated deposition events were generated over 7 successive days by resuspending the sediments at 4.00 pm each day. Deposition events were thus scheduled to occur at night to avoid attenuation of light by suspended sediments, which is a key dredging related pressure impacting upon coral health^104^, and therefore deposition at night reducing the confounding nature of extra causal pathways impacting on health that were not investigated here.

Sediment was delivered to individual tanks by an air diaphragm pump (SandPiper S1F), via a high velocity loop (3 m s^−1^), from a concentrated (g L^−1^) stock solution. The desired SSC in each tank was controlled by a programmable logic controller (PLC) coupled to pivoting solenoid valves connected to the stock solution, and based on real time feedback from turbidity sensors (Turbimax CUS31, Endress and Hauser) contained in each of the 12 tanks.

Sediment deposition was measured within each tank using sediment pods (SedPods), which are concrete filled PVC cylinders^105^ placed on the grating beside the corals. Each morning SedPods were capped and any accumulated sediment filtered through pre-weighed 0.4 µm, 47 mm diameter polycarbonate filters, incubated at 60 °C for ≥24 h, and weighed to determine sediment mass.

All sediment used was biogenic calcium carbonate collected from Davies Reef (GBRMPA permit: G13/35758.1). Sediment was first screened to 2 mm and then ground with a rod mill grinder until the mean grain size was ~30 µm (range: 0.5–140 µm), measured using laser diffraction techniques (Mastersizer 2000, Malvern instruments Ltd, UK). Total organic content of the sediment was 0.25% (w/w).

For each sediment treatment, there were 3 replicate tanks with 4 normally-pigmented and 4 bleached corals per tanks. Corals were exposed to 7 individual deposition events (one per day). Coral fragments were photographed immediately before the first deposition event and immediately after the last. Changes in the colour of coral tissue was used as a non-destructive indicator of bleaching. The photographs were analysed with Image J^106^ using the histogram function on a selection of representative live tissue, taking the arithmetic mean of pixel values (range 0–255) on a black and white scale. These were then standardised by converting to a range between 0 and 1 for each species. During the photographing process, any partial mortality of the corals was noted and then quantified from the photographs using image processing software Image J.

Chlorophyll fluorescence of the endosymbiotic dinoflagellate microalgae within tissue of each coral fragment was measured using a mini-PAM fluorometer (Walz, Germany). Measurements were obtained using a 6 mm fibre-optic probe positioned perpendicular to the coral fragment and 3 mm away (controlled by a rubber spacer). Coral fragments were dark-adapted for 30 min prior to measuring initial fluorescence (*F*_0_), which was determined by applying a weak pulse-modulated red light (650 nm, ~0.15 μmol photons m^−2^ s^−1^). Maximum fluorescence (*F*_m_) was then measured following a saturating pulse of saturating white light. Maximum quantum yield (*F*_*v*_/*F*_*m*_) is the proportion of light used for photosynthesis by chlorophyll when all reaction centres are open^107^ and is determined by the following equation:

$$\frac{{F}_{{\rm{v}}}}{{F}_{{\rm{m}}}}=({F}_{{\rm{m}}}-{F}_{0})/{F}_{{\rm{m}}}$$

Chlorophyll fluorescence measurements were made at the start of the sedimentation experiments and again at the end. Measurements were only taken over live tissue, with 4 measurements taken and averaged per coral replicate.

The mass of sediment that remained on coral tissue after it was left to settle overnight (~16 h) was quantified after both the first and last deposition event. To do this a plastic bag was placed around each coral and the bag then sealed and shaken to dislodge all sediment that had settled on the surfaces. The suspension was then filtered through pre-weighed 47 mm diameter polycarbonate filters (0.4 µm nominal pore size), dried at 60 °C for ≥24 h, and weighed to 0.0001 g. The mass of collected sediment was then standardised to fragment surface area using the wax dipping technique^108^.

At the end of the experiment, one third of the corals were snap frozen in liquid nitrogen and then stored at −80 °C. Fragments were then air blasted to remove tissue and chlorophyll extracted twice from tissue using 95% ethanol, and quantified spectroscopically using the equations of Ritchie^109^ and Lichtenthaler^110^. Endolithic algae were not visible in the samples and assumed not to contribute any significant amount of Chlorophyll a (Chl *a*) to the samples. Chl *a* was standardised to the surface area and used as a proxy for coral bleaching.

All data were analysed with R software (version 3.2.3, R Core Team^111^). We used a complete subsets model selection approach^112^ to examine the effects of time (1–7 days of repeated exposure), sedimentation level (0, 11, 22 and 40 mg cm^−2^ d^−1^), species (*A*. *millepora*, *T*. *reniformis* and *Porites spp*.), and bleaching status (bleached, normally pigmented) on coral health parameters. This approach involves fitting all possible model combinations (including interactions) and comparing this complete model set using Akaike Information Criterion with corrections for small sample sizes (AICc). The model with the lowest AICc (within 2) and the fewest parameters is considered the most parsimonious, and chosen as the best model. For modelling of relationships, tank and coral fragment identity (to account for repeated measurements through time) were included as random factors in a Generalised Linear Mixed Model (GLMM). A complete subsets information theoretic approach based on GLMM was used over more traditional ANOVA like approaches, as this allows non-independence (associated with repeated measurements through time and tank effects) to be successfully accommodated, as well as flexibility of the statistical distributions used. Colour index and *F*_*v*_/*F*_*m*_ were logit transformed, as they are both proportions, and then modelled using a Gaussian distribution, with bleaching status, time, sediment deposition treatment and species included as fixed factors. Models were fit using the lmer function from the package lme4^113^. Chl *a* concentrations, and the amount of deposited sediment remaining on coral fragments after the 1st and 7th deposition event, were modelled as GLMMs based on a Tweedie distribution, fit using the cplm package, with fragment number and tank included as random effects^114^. A Tweedie distribution is appropriate for continuously distributed data that can take values of 0 to ∞.

Due to the interaction between time and species on the amount of deposited sediment remaining on coral fragments, pressure-response relationships were subsequently fitted after 1 and 7 deposition events, for both bleached and normally pigmented fragments. These relationships were fitted with the drc package^115^.

## Electronic supplementary material

Supplimentary Information

## References

[1] Carpenter KE (2008). One-third of reef-building corals face elevated extinction risk from climate change and local impacts. Science.

[2] Gardner TA, Côté IM, Gill JA, Grant A, Watkinson AR (2003). Long-term region-wide declines in Caribbean corals. Science.

[3] Hoegh-Guldberg O (1999). Climate change, coral bleaching and the future of the world’s coral reefs. Mar. Freshwat. Res.

[4] Hughes TP (2003). Climate change, human impacts, and the resilience of coral reefs. Science.

[5] Knowlton N, Jackson JB (2008). Shifting baselines, local impacts, and global change on coral reefs. PLoS Biol..

[6] Wilkinson, C. R. Coral reefs of the world are facing widespread devastation: Can we prevent this through sustainable management practices? *Proceedings of the 7th International Coral Reef Symposium*. (1992).

[7] Wilkinson CR (1999). Global and local threats to coral reef functioning and existence: review and predictions. Mar. Freshwat. Res.

[8] Darling ES, McClanahan TR, Côté I (2010). Combined effects of two stressors on Kenyan coral reefs are additive or antagonistic, not synergistic. Conservation Letters.

[9] Donner SD, Skirving WJ, Little CM, Oppenheimer M, Hoegh‐Guldberg O (2005). Global assessment of coral bleaching and required rates of adaptation under climate change. Global Change Biol.

[10] Hoegh-Guldberg O (2007). Coral reefs under rapid climate change and ocean acidification. Science.

[11] Anthony, K. Ecosystem damages from loss of coral reefs under climate change. *Annu Rev Environ Resour* **41** (2016).

[12] Halpern BS, McLeod K, Rosenberg A, Crowder L (2008). Managing for cumulative impacts in ecosystem-based management through ocean zoning. Ocean Coast Management.

[13] Halpern B (2008). A Global Map of Human Impact on Marine Ecosystems. Science.

[14] Côté IM, Darling ES, Brown CJ (2016). Interactions among ecosystem stressors and their importance in conservation. Proceedings of the Royal Society B: Biological Sciences.

[15] Marshall, P. & Schuttenburg, H. A reef manager’s guide to coral bleaching. *Great Barrier Reef Marine Park Authority* (2006).

[16] Brown B (1997). Coral bleaching: causes and consequences. Coral Reefs.

[17] Brown B, Howard L (1985). Assessing the effects of” stress” on reef corals. Adv. Mar. Biol.

[18] Jones R (1997). Zooxanthellae loss as a bioassay for assessing stress in corals. Mar. Ecol. Prog. Ser..

[19] Douglas A (2003). Coral bleaching–how and why?. Mar. Pollut. Bull..

[20] Glynn PW (1996). Coral reef bleaching: facts, hypotheses and implications. Global Change Biol.

[21] Jones R (2008). Coral bleaching, bleaching-induced mortality, and the adaptive significance of the bleaching response. Mar. Biol.

[22] Eakin, M. *et al*. Global Coral Bleaching 2014–2017? 6pp. (Reef Encounter 6pp., 2016).

[23] Berkelmans R, De’Ath G, Kininmonth S, Skirving W (2004). A comparison of the 1998 and 2002 coral bleaching events on the Great Barrier Reef: spatial correlation, patterns, and predictions. Coral Reefs.

[24] Goreau TF, McClanahan TR, Hayes RL, Strong A (2000). Conservation of coral reefs after the 1998 global bleaching event. Conserv. Biol..

[25] Heron S, Maynard J, van Hooidonk R, Eakin CM (2016). Warming trends and bleaching stress of the world’s coral reefs 1985–2012. Scientific Reports.

[26] Fitt WK, McFarland FK, Warner ME, Chilcoat GC (2000). Seasonal patterns of tissue biomass and densities of symbiotic dinoflagellates in reef corals and relation to coral bleaching. Limnol. Oceanogr..

[27] Gilmour JP, Smith LD, Heyward AJ, Baird AH, Pratchett MS (2013). Recovery of an Isolated Coral Reef System Following Severe Disturbance. Science.

[28] Edmunds P (1994). Evidence that reef-wide patterns of coral bleaching may be the result of the distribution of bleaching-susceptible clones. Mar. Biol.

[29] Marshall P, Baird AH (2000). Bleaching of corals on the Great Barrier Reef: differential susceptibilities among taxa. Coral Reefs.

[30] Cunning R, Ritson-Williams R, Gates RD (2016). Patterns of bleaching and recovery of *Montipora capitata* in Kāneohe Bay, Hawaii, *USA*. Mar. Ecol. Prog. Ser..

[31] Edmunds PJ, Gates RD, Gleason DF (2003). The tissue composition of *Montastraea franksi* during a natural bleaching event in the Florida Keys. Coral Reefs.

[32] Fitt WK, Spero HJ, Halas J, White MW, Porter JW (1993). Recovery of the coral Montastrea annularis in the Florida Keys after the 1987 Caribbean “bleaching event”. Coral Reefs.

[33] Jokiel PL, Coles SL (1990). Response of Hawaiian and other Indo-Pacific reef corals to elevated temperature. Coral Reefs.

[34] Jones R, Yellowlees D (1997). Regulation and control of intracellular algae (=zooxanthellae) in hard corals. Philosophical Transactions of the Royal Society of London B: Biological Sciences.

[35] Grottoli AG, Rodrigues LJ, Juarez C (2004). Lipids and stable carbon isotopes in two species of Hawaiian corals, *Porites compressa* and *Montipora verrucosa*, following a bleaching event. Mar. Biol.

[36] Grottoli AG, Rodrigues LJ, Palardy JE (2006). Heterotrophic plasticity and resilience in bleached corals. Nature.

[37] Grottoli AG (2014). The cumulative impact of annual coral bleaching can turn some coral species winners into losers. Global Change Biol.

[38] Rodrigues LJ, Grottoli AG (2007). Energy reserves and metabolism as indicators of coral recovery from bleaching. Limnol. Oceanogr..

[39] Schoepf, V. *et al*. Annual coral bleaching and the long-term recovery capacity of coral. *Proceedings of the Royal Society B: Biological Sciences* **282**, doi:10.1098/rspb.2015.1887 (2015). 10.1098/rspb.2015.1887 PMC4685810 26582020

[40] Anthony KRN, Hoogenboom MO, Maynard JA, Grottoli AG, Middlebrook R (2009). Energetics approach to predicting mortality risk from environmental stress: a case study of coral bleaching. Funct. Ecol..

[41] Glynn PW, Peters EC, Muscatine L (1985). Coral tissue microstructure and necrosis: relation to catastrophic coral mortality in Panama. Dis. Aquat. Org..

[42] Lasker HR, Peters EC, Coffroth MA (1984). Bleaching of reef coelenterates in the San Blas Islands, Panama. Coral Reefs.

[43] Hayes RL, Bush P (1990). Microscopic observations of recovery in the reef-building scleractinian coral, *Montastrea annularis*, after bleaching on a Cayman reef. Coral Reefs.

[44] Fitt W (2009). Response of two species of Indo-Pacific corals, *Porites cylindrica* and *Stylophora pistillata*, to short-term thermal stress: the host does matter in determining the tolerance of corals to bleaching. J. Exp. Mar. Biol. Ecol..

[45] Piggot AM, Fouke BW, Sivaguru M, Sanford RA, Gaskins HR (2009). Change in zooxanthellae and mucocyte tissue density as an adaptive response to environmental stress by the coral, *Montastraea annularis*. Mar. Biol.

[46] Gates RD, Baghdasarian G, Muscatine L (1992). Temperature stress causes host cell detachment in symbiotic cnidarians: implications for coral bleaching. The Biological Bulletin.

[47] Bhagooli R, Hidaka M (2004). Release of zooxanthellae with intact photosynthetic activity by the coral *Galaxea fascicularis* in response to high temperature stress. Mar. Biol.

[48] Fitt WK, Brown BE, Warner ME, Dunne RP (2001). Coral bleaching: interpretation of thermal tolerance limits and thermal thresholds in tropical corals. Coral Reefs.

[49] Mendes JM, Woodley JD (2002). Timing of reproduction in *Montastraea annularis*: relationship to environmental variables. Mar. Ecol. Prog. Ser..

[50] Szmant AM, Gassman NJ (1990). The effects of prolonged “bleaching” on the tissue biomass and reproduction of the reef coral *Montastrea annularis*. Coral Reefs.

[51] Porter J, Fitt W, Spero H, Rogers C, White I (1989). Bleaching in reef corals: physiological and stable isotopic responses. Proc. Natl. Acad. Sci. USA.

[52] Bessell-Browne P, Stat M, Thomson D, Clode PL (2014). *Coscinaraea marshae* corals that have survived prolonged bleaching exhibit signs of increased heterotrophic feeding. Coral Reefs.

[53] Diaz-Pulido G, McCook L (2002). The fate of bleached corals: patterns and dynamics of algal recruitment. Mar. Ecol. Prog. Ser..

[54] Bruno JF, Selig ER (2007). Regional decline of coral cover in the Indo-Pacific: timing, extent, and subregional comparisons. PLOS ONE.

[55] Manzello DP (2015). Rapid recent warming of coral reefs in the Florida Keys. Scientific reports.

[56] Miller MW (2016). Detecting sedimentation impacts to coral reefs resulting from dredging the Port of Miami, Florida USA. PeerJ.

[57] Chin, A. & Marshall, P. Reactive monitoring at Nelly Bay Harbour using environmental monitoring to manage marine construction activities. (Australian Institute of Marine Science and the IUCN Marine Program, 2003).

[58] Hanley JR (2011). Environmental monitoring programs on recent capital dredging projects in the Pilbara (2003–10): a review. APPEA.

[59] EPA. Gorgon Gas Development Revised and Expanded Proposal: Barrow Island nature reserve. Report and recommendations of the Environmental Protection Authority. Bulletin 1221. 280 pp. (Environmental Protection Authority (EPA), Perth, Western Australia, 2006).

[60] EPA. Pluto LNG development, Burrup Peninsula. Report and recommendations of the Environmental Protection Authority. Bulletin 1259. 406 pp. (Environmental Protection Authority (EPA), Perth, Western Australia, 2007).

[61] EPA (2011). Environmental Assessment Guidleine for Marine Dredging Programs EAG7.

[62] Depczynski M (2013). Bleaching, coral mortality and subsequent survivorship on a West Australian fringing reef. Coral Reefs.

[63] Feng M, McPhaden MJ, Xie S-P, Hafner J (2013). La Niña forces unprecedented Leeuwin Current warming in 2011. Scientific reports.

[64] Lafratta A, Fromont J, Speare P, Schönberg C (2016). Coral bleaching in turbid waters of north-western Australia. Mar. Freshwat. Res.

[65] Moore JAY (2012). Unprecedented mass bleaching and loss of coral across 12° of latitude in Western Australia in 2010–11. PLoS One.

[66] Foster, T. *et al*. Dredging and port construction around coral reefs. *PIANC Environmental Commission*, *Report No* **108** (2010).

[67] VBKO. Protocol for the Field Measurementof Sediment Release from Dredgers. A practical guide to measuring sediment release from dredging plant for calibration and verification of numerical models. 1–83 pp. (Reporrt produced for VBKO TASS project by HR Wallingford Ltd & Dredging Research Ltd Issue, 2003).

[68] Jones R, Bessell-Browne P, Fisher R, Klonowski W, Slivkoff M (2016). Assessing the impacts of sediments from dredging on corals. Mar. Pollut. Bull..

[69] ISRS. The effects of terrestrial runoff of sediments, nutrients and other pollutants on coral reefs. Briefing Paper 3, International Society for Reef Studies. 18 pp. (2004).

[70] Erftemeijer PL, Riegl B, Hoeksema BW, Todd PA (2012). Environmental impacts of dredging and other sediment disturbances on corals: a review. Mar. Pollut. Bull..

[71] Jones R, Fisher R, Stark C, Ridd P (2015). Temporal patterns in seawater quality from dredging in tropical environments. PLoS One.

[72] Rogers CS (1990). Responses of coral reefs and reef organisms to sedimentation. Mar. Ecol. Prog. Ser..

[73] Berkelmans R (2002). Time-integrated thermal bleaching thresholds of reefs and their variation on the Great Barrier Reef. Mar. Ecol. Prog. Ser..

[74] Berkelmans R, Willis BL (1999). Seasonal and local spatial patterns in the upper thermal limits of corals on the inshore Central Great Barrier Reef. Coral Reefs.

[75] Hoegh-Guldberg O, Jones R (1999). Photoinhibition and photoprotection in symbiotic dinoflagellates from reef-building corals. Mar. Ecol. Prog. Ser..

[76] Iglesias-Prieto R, Matta JL, Robins WA, Trench RK (1992). Photosynthetic response to elevated temperature in the symbiotic dinoflagellate *Symbiodinium* microadriaticum in culture. Proc. Natl. Acad. Sci. USA.

[77] Jones R, Hoegh-Guldberg O, Larkum A, Schreiber U (1998). Temperature‐induced bleaching of corals begins with impairment of the CO_2_ fixation mechanism in zooxanthellae. Plant, Cell Environ.

[78] Jones R, Hoegh-Guldberg O (2001). Diurnal changes in the photochemical efficiency of the symbiotic dinoflagellates (Dinophyceae) of corals: photoprotection, photoinactivation and the relationship to coral bleaching. Plant, Cell Environ.

[79] Warner ME, Fitt W, Schmidt G (1996). The effects of elevated temperature on the photosynthetic efficiency of zooxanthellae in hospite from four different species of reef coral: a novel approach. Plant, Cell Environ.

[80] Warner ME, Fitt WK, Schmidt GW (1999). Damage to photosystem II in symbiotic dinoflagellates: A determinant of coral bleaching. Proc. Natl. Acad. Sci. USA.

[81] Duerden JE (1906). The role of mucus in corals. Q J Microsc Sci.

[82] Hubbard JA, Pocock YP (1972). Sediment rejection by recent scleractinian corals: a key to palaeo-environmental reconstruction. Geologische Rundschau.

[83] Marshall, S. M. & Orr, A. P. *Sedimentation on Low Isles Reef and its relation to coral growth*. (British Museum (Natural History), 1931).

[84] Rogers CS (1983). Sublethal and lethal effects of sediments applied to common Caribbean reef corals in the field. Mar. Pollut. Bull..

[85] Stafford-Smith M, Ormond R (1992). Sediment-rejection mechanisms of 42 species of Australian scleractinian corals. Mar. Freshwat. Res.

[86] Weber M, Lott C, Fabricius KE (2006). Sedimentation stress in a scleractinian coral exposed to terrestrial and marine sediments with contrasting physical, organic and geochemical properties. J. Exp. Mar. Biol. Ecol..

[87] Weber M (2012). Mechanisms of damage to corals exposed to sedimentation. Proc. Natl. Acad. Sci. USA.

[88] Whinney, J., Jones, R., Duckworth, A. & Ridd, P. Continuous *in situ* monitoring of sediment deposition in shallow benthic environments. *Coral Reefs*, 1–13, doi:10.1007/s00338-016-1536-7 (2017).

[89] Brown B, Bythell J (2005). Perspectives on mucus secretion in reef corals. Mar. Ecol. Prog. Ser..

[90] Bythell JC, Wild C (2011). Biology and ecology of coral mucus release. J. Exp. Mar. Biol. Ecol..

[91] Edmunds PJ, Davies PS (1989). An energy budget for *Porites porites* (Scleractinia), growing in a stressed environment. Coral Reefs.

[92] Riegl, B. & Branch, G. Effects of sediment on the energy budgets of four scleractinian (Bourne 1900) and five alcyonacean (Lamouroux 1816) corals. *J*. *Exp*. *Mar*. *Biol*. *Ecol*. **186**, 259–275 (1995).

[93] Bak R, Elgershuizen J (1976). Patterns of oil-sediment rejection in corals. Mar. Biol.

[94] Stafford-Smith MG (1993). Sediment-rejection efficiency of 22 species of Australian scleractinian corals. Mar. Biol.

[95] Shapiro OH (2014). Vortical ciliary flows actively enhance mass transport in reef corals. Proc. Natl. Acad. Sci. USA.

[96] Anthony K (2000). Enhanced particle-feeding capacity of coral on turbid reefs (Great Barrier Reef, Australia). Coral Reefs.

[97] Mills M, Sebens K (1997). Particle ingestion efficiency of the corals *Siderastrea siderea* and *Agaricia agaricites*: Effects of flow speed and sediment loads. 8th Int Coral Reef Symp.

[98] Bessell-Browne P, Fisher R, Duckworth A, Jones R (2017). Mucous sheet production in Porites: an effective bioindicator of sediment related pressures. Ecol. Indicators.

[99] Ports Australia. Dredging and Australian ports. 0–94 (Sydney, NSW, Australia 2014).

[100] EPA. Technical Guidance: Environmental Impact Assessment of Marine Dredging Proposals. (Environmental Protection Authority, Perth, Western Australia, 2016).

[101] Suedel BC, Kim J, Clarke DG, Linkov I (2008). A risk-informed decision framework for setting environmental windows for dredging projects. Science of The Total Environment.

[102] PIANC. Dredging and Port Construction around Coral Reefs., 75pp. (The World Association for Waterborne Transport Infrastructure (PIANC), 2010).

[103] Veron, J. Corals of the World. Vol 1–3 (2000).

[104] Bessell-Browne, P. *et al*. Impacts of turbidity on corals: The relative importance of light limitation and suspended sediments. *Mar*. *Pollut*. *Bull*., doi:10.1016/j.marpolbul.2017.01.050 (2017). 10.1016/j.marpolbul.2017.01.050 28162249

[105] Field ME, Chezar H, Storlazzi CD (2012). SedPods: a low-cost coral proxy for measuring net sedimentation. Coral Reefs.

[106] Schneider CA, Rasband WS, Eliceiri KW (2012). NIH Image to ImageJ: 25 years of image analysis. Nature methods.

[107] Genty B, Briantais J-M, Baker NR (1989). The relationship between the quantum yield of photosynthetic electron transport and quenching of chlorophyll fluorescence. Biochim. Biophys, Acta.

[108] Stimson J, Kinzie RA (1991). The temporal release of zooxanthellae from the reef coral *Pocillopora damicornis* (Linnaeus) under nitrogen-enrichment and control conditions. J. Exp. Mar. Biol. Ecol..

[109] Ritchie R (2008). Universal chlorophyll equations for estimating chlorophylls a, b, c, and d and total chlorophylls in natural assemblages of photosynthetic organisms using acetone, methanol, or ethanol solvents. Photosynthetica.

[110] Lichtenthaler HK (1987). Chlorophyll and carotenoids: pigments of photosynthetic biomembranes. Methods in Enzymology.

[111] R: A language and envrionment for statistical computing (R Foundation for statistical computing, Vienna, Austria, 2015).

[112] Burnham, K. P. & Anderson, D. R. Model Selection and Multimodel Inference: A Practical Information-Theoretic Approach. (Springer, 2002).

[113] Bates D, Maechler M, Bolker B, Walker S (2015). Fitting linear mixed-effects models using lme4. J Stat Softw.

[114] Zhang Y (2013). Likelihood-based and Bayesian Methods for Tweedie Compound Poisson Linear Mixed Models. Statistics and Computing.

[115] Ritz C, Baty F, Streibig JC, Gerhard D (2015). Dose-Response Analysis Using R. PLoS One.

