# Supplementary material for: Cumulative impacts: thermally bleached corals have reduced capacity to clear deposited sediment
Source: Sci Rep. 2017 Jun 2;7:2716. doi: 10.1038/s41598-017-02810-0 (PMC5457406; doi:10.1038/s41598-017-02810-0)
Supplement: Supplementary file 1 — Supplimentary Information [file 41598_2017_2810_MOESM1_ESM.pdf]

Cumulative impacts: thermally bleached corals have reduced capacity to clear deposited sediment

P. Bessell-Browne, A. P. Negri, R. Fisher, P. L. Clode, R. Jones

Supplementary Information

Table S1. The amount of sediment (mg cm^{-2}, mean ± SE, *n*=9) remaining on normally-pigmented and bleached coral fragments after 1 and 7 deposition events.

		After 1 deposition event		After 7 deposition events	
Species	Mean SedPod (mg cm^{-2})	Normally pigmented	Bleached	Normally pigmented	Bleached
Acropora millepora	0	0.0 ± 0.0	0.0 ± 0.0	0.1 ± 0.0	0.2 ± 0.2
	11	0.1 ± 0.0	0.2 ± 0.0	1.3 ± 0.4	4.4 ± 1.5
	22	0.3 ± 0.1	0.3 ± 0.1	1.6 ± 0.6	9.6 ± 6.1
	40	0.3 ± 0.1	0.5 ± 0.2	2.4 ± 0.9	10.7 ± 4.1
Porites spp.	0	0.0 ± 0.0	0.0 ± 0.0	0.0 ± 0.0	0.0 ± 0.0
	11	0.3 ± 0.2	0.3 ± 0.1	0.6 ± 0.2	1.2 ± 0.5
	22	0.6 ± 0.3	2.6 ± 2.0	3.9 ± 2.8	4.6 ± 3.1
	40	0.7 ± 0.4	3.3 ± 1.9	2.8 ± 2.1	12.3 ± 7.7
Turbinaria reniforms	0	0.1 ± 0.1	0.0 ± 0.0	0.1 ± 0.0	0.0 ± 0.0
	11	0.2 ± 0.1	0.2 ± 0.1	0.8 ± 0.4	1.1 ± 1.2
	22	0.5 ± 0.1	0.8 ± 0.3	1.0 ± 0.5	4.4 ± 3.6
	40	0.7 ± 0.2	1.5 ± 0.9	2.3 ± 1.0	4.9 ± 2.5

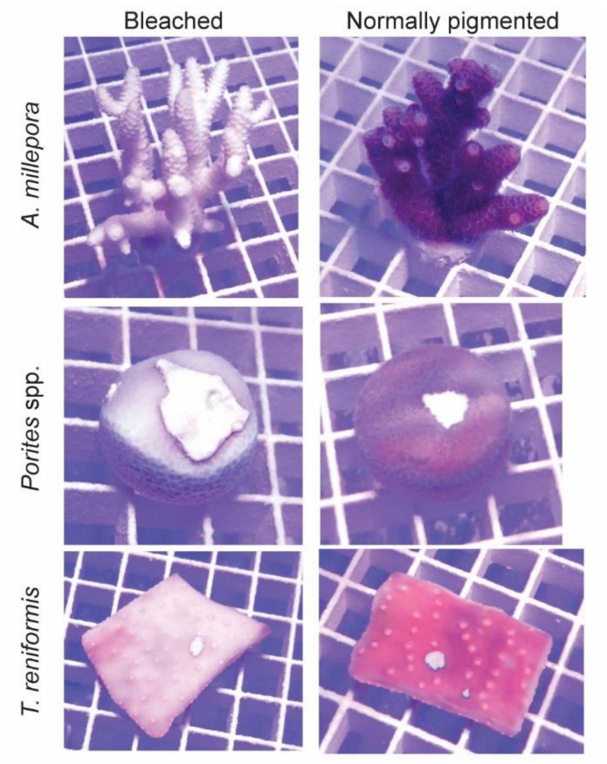

Figure S1. Photographs of representative colonies of each of the three species, *A. millepora*, *Porites* spp. and *T. reniformis* that were bleached or normally pigmented in the 40 mg cm^{-2} d^{-1} deposition treatment.
